# Low levels of HIV test coverage in clinical settings in the UK: a systematic review of adherence to 2008 guidelines

**DOI:** 10.1136/sextrans-2013-051312

**Published:** 2014-01-10

**Authors:** Rahma Elmahdi, Sarah M Gerver, Gabriela Gomez Guillen, Sarah Fidler, Graham Cooke, Helen Ward

**Affiliations:** 1 Department of Infectious Disease Epidemiology, Imperial College London, London, UK; 2 Department of Global Health, Academic Medical Center, University of Amsterdam and Amsterdam Institute for Global Health and Development, Amsterdam, Netherlands; 3 Department of Communicable Diseases, Department of Medicine,Imperial College London, London, UK

**Keywords:** HIV TESTING, HIV, DIAGNOSIS

## Abstract

**Objectives:**

To quantify the extent to which guideline recommendations for routine testing for HIV are adhered to outside of genitourinary medicine (GUM), sexual health (SH) and antenatal clinics.

**Methods:**

A systematic review of published data on testing levels following publication of 2008 guidelines was undertaken. Medline, Embase and conference abstracts were searched according to a predefined protocol. We included studies reporting the number of HIV tests administered in those eligible for guideline recommended testing. We excluded reports of testing in settings with established testing surveillance (GUM/SH and antenatal clinics). A random effects meta-analysis was carried out to summarise level of HIV testing across the studies identified.

**Results:**

Thirty studies were identified, most of which were retrospective studies or audits of testing practice. Results were heterogeneous. The overall pooled estimate of HIV test coverage was 27.2% (95% CI 22.4% to 32%). Test coverage was marginally higher in patients tested in settings where routine testing is recommended (29.5%) than in those with clinical indicator diseases (22.4%). Provider test offer was found to be lower (40.4%) than patient acceptance of testing (71.5%).

**Conclusions:**

Adherence to 2008 national guidelines for HIV testing in the UK is poor outside of GUM/SH and antenatal clinics. Low levels of provider test offer appear to be a major contributor to this. Failure to adhere to testing guidelines is likely to be contributing to late diagnosis with implications for poorer clinical outcomes and continued onwards transmission of HIV. Improved surveillance of HIV testing outside of specialist settings may be useful in increasing adherence testing guidelines.

## Introduction

In 2011, there were an estimated 96 000 people living with HIV in the UK with almost one in four thought to be unaware of their infection.^1^ In the same year, 6280 individuals were newly diagnosed and 47% of these cases were at a late stage of infection (CD4 cell count <350 cells/mm^3^).^1^ Patients diagnosed late experience a higher risk of developing AIDS and a tenfold increased risk of death within a year of diagnosis.^2–4^ Timely identification of those who are HIV-positive and appropriate referral into care services is essential not only for the reduction of HIV-associated morbidity and mortality but also for the prevention of onwards transmission of the virus. Reducing late HIV diagnosis is a key indicator of the Public Health Outcomes Framework set by the Department of Health (DoH),^5^ and the primary means of achieving this is widespread testing in populations and settings at increased risk of HIV infection.

The latest national guidelines on HIV testing were published in October 2008. The guidelines were published by the British HIV Association (BHIVA) and written in collaboration with the British Infection Society (BIS) and the British Association for Sexual Health and HIV (BASHH). These guidelines were intended to promote an increase in HIV testing in all healthcare settings to reduce the proportion of individuals with undiagnosed HIV infection. The authors of the guidelines state the reason for the need of their publication as being (a) misconceptions regarding HIV testing remaining a hindrance to increased testing; (b) the importance of both the individual patient and public health benefits of increased testing and (c) the need for up-to-date guidance that would enable any clinician to perform an HIV test within good clinical practice, thereby encouraging the ‘normalisation’ of HIV testing.^6^ These guidelines recommended HIV testing in a wider range of clinical settings and populations including those with indicator diseases, all medical admissions and new registrants in primary care in areas with a diagnosed adult HIV prevalence of greater than 2 per 1000 population (please see online appendix a, supplementary data). The guidelines have additionally been endorsed by the National Institute of Health and Care Excellence and incorporated into their pathway for strategy, policy and commissioning on HIV testing and prevention.^7^ ^8^

There have been improvements in earlier diagnosis for HIV in the UK. According to Public Health England figures, there has been a gradual decrease in the proportion of people diagnosed late with HIV in the UK, from 60% in 2002 to 47% in 2011^1^; however, this remains high, suggesting that testing programmes continue to miss people at an early stage in their infection. HIV testing is routinely monitored in genitourinary medicine (GUM), sexual health (SH) and antenatal clinics (ANC) where uptake is high, with 70% of GUM and 97% of ANC attendees being tested for HIV in 2010; these locations account for 47% and 31% of total HIV tests in the UK, respectively. However, there is no routine monitoring of testing in other (non-specialist) clinical settings or populations, and therefore adherence to national guidelines is unknown. To inform decision making about future HIV-testing initiatives, we reviewed evidence of adherence to national guidelines in settings not covered by existing surveillance.

## Methods

### Search strategy and inclusion/exclusion criteria

We carried out a systematic review and meta-analysis on levels of adherence to national guideline recommended HIV testing in non-specialist settings. A predefined protocol (available as online supplementary file ‘Review Protocol’) detailing inclusion and exclusion criteria was developed; two authors (RE and SMG) independently used a set combination of terms (HIV, human immunodeficiency virus test*, screen*, diagnos*, United Kingdom, UK, England, Northern Ireland, Scotland, Wales, Britain, British, English, Scottish, Welsh, Northern Irish) to search MEDLINE, Embase, Maternity and Infant Care and PsychINFO databases via the search engine Ovid. The final search was run on 28 February 2013. In addition, bibliographies from eligible papers, conference abstracts and grey literature (including relevant reports^7^ ^8^) were hand searched. Studies were included if they measured HIV test coverage in a defined, eligible population. Studies were excluded if they related to testing in GUM/SH or ANC (specialist) clinics, included data from before September 2008 or were conducted outside the UK. Studies not measuring HIV testing levels as an outcome were also excluded as were those measuring HIV testing in community settings as, although testing in these settings is encouraged, it is not explicitly recommended in UK national guidelines. In order to identify as wide a range of studies measuring HIV testing levels as possible, all quantitative study designs and methodologies were included. Where key information for article inclusion was missing, an online search for conference presentations/posters was performed and authors were contacted for additional data. Articles were only excluded after the deadline period for author reply had passed.

### Data extraction

Data extraction was undertaken separately by two authors, and information on the following variables was retrieved: author(s), exposure status or risk group (if applicable), primary HIV testing outcome (how receipt of HIV test was confirmed), exclusion criteria, time period and duration of data collection, population, setting (and Public Health England estimations for diagnosed HIV prevalence per 1000 population 15–59 year olds), type and number of centres, study design and methods, measure or reporting method, type of test used, method of service delivery, opt-in/opt-out model, number of patients eligible for testing, number offered testing, number tested and number with positive test result.

### Data analysis

Studies were classified into two groups according to patient population or setting where testing took place: persons diagnosed with a disease indicative of HIV infection and persons attending a setting where routine HIV screening should be undertaken (excluding GUM/SH and ANC settings) (see online appendix a, supplementary data).^5^ Test coverage, defined as the percentage of those eligible for HIV testing who were offered and accepted an HIV test, was calculated for each study identified. Additional outcomes including (a) test offer level defined as the percentage of those eligible for testing who were offered a test), (b) test acceptance level (defined as the percentage of those offered an HIV test who were tested) and (c) seroprevalence level (defined as the percentage of those testing positive for HIV) were calculated where this information was available. Using a random effects model, stratified analyses were performed by group. Clopper–Pearson 95% CIs were calculated for each study input. CIs for these results were capped at 0% and 100% for presentation of pooled estimates as percentages.^8^ Cochran's test of heterogeneity (Q statistic) and I^2^ statistic was used to assess the presence of and quantify the extent of between-study heterogeneity in testing prevalence estimates.^9^

Univariate meta-regression was used to investigate heterogeneity in overall testing coverage. There were too few studies to explore this for the other outcomes. Proportions were transformed to logits using a continuity correction of 0.1% where the number of patients tested for HIV was either equal to zero or the number of eligible patients.^10^ If a covariate was significantly associated with the prevalence estimates, the percentage of between-study variability explained by the covariate (R^2^) was calculated as 100*(1− (**τ**^2^ regression model with covariate/ **τ**^2^ regression without covariate)). Analyses were completed in STATA v.11.0 (StataCorp, College station, Texas, USA).

## Results

**Figure 1:**
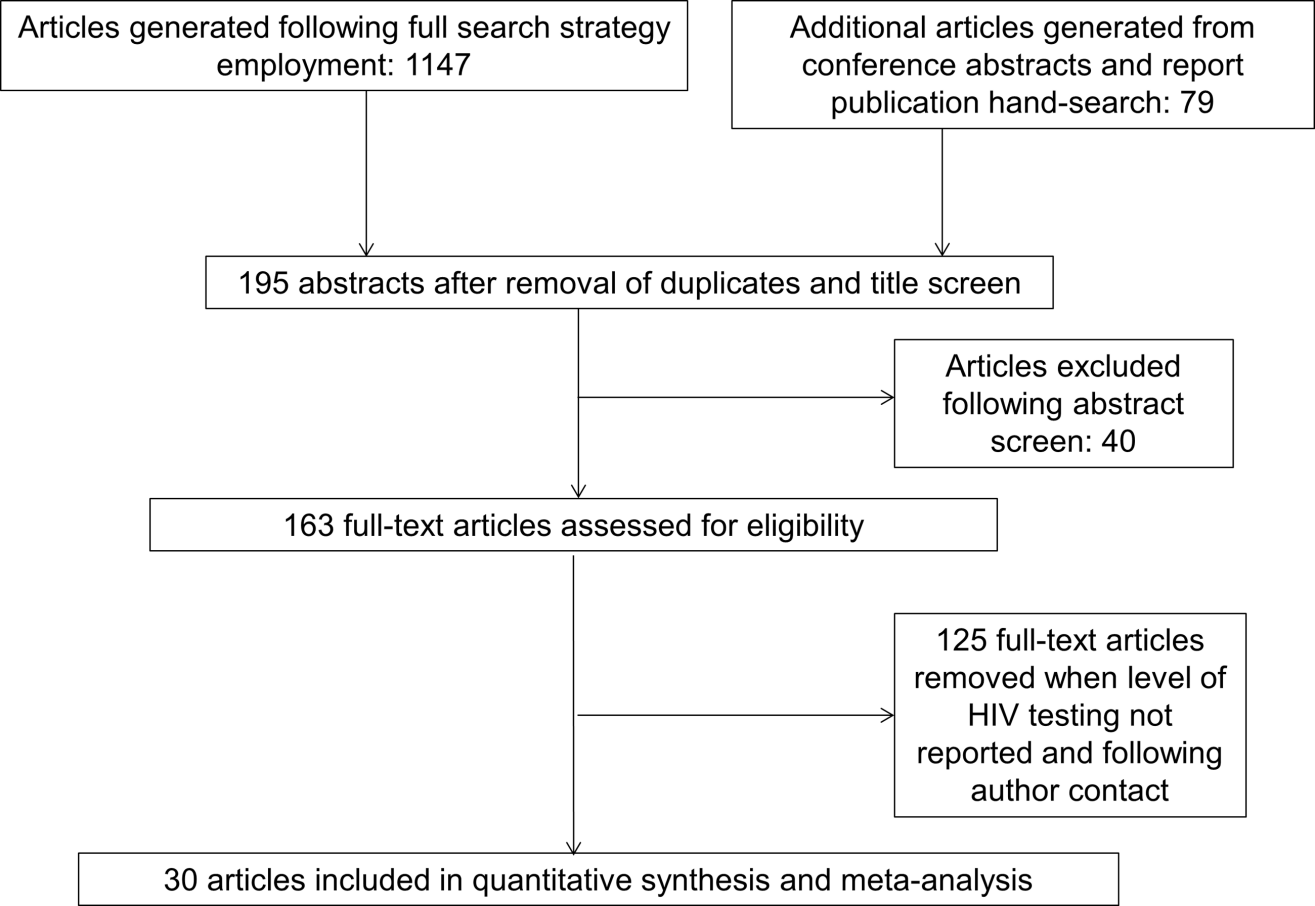
Flowchart of search results and selection of papers.

The search identified 1226 references that were screened; after exclusion of duplicates and undertaking a title and abstract screen, 163 full-text articles were evaluated for full inclusion. Of these, 30 reports that measured levels of HIV testing in a range of recommended settings were identified (figure 1). Fourteen were cross-sectional studies or retrospective studies (audits) from hospital settings using either case note review or extraction of data from electronic or paper records. Data from 12 were in journal publications, and data from the remaining 18 studies were extracted from published reports or conference abstracts. Ten studies were in patients diagnosed with an indicator disease and 20 in people attending services where routine HIV testing was recommended due to diagnosed prevalence in the local population. Information for all 30 studies identified can be found in online supplementary data file appendix b: characteristics of studies included: methods, measures and testing levels and appendix c: supplementary data tables for studies identified by group.

**Figure 2:**
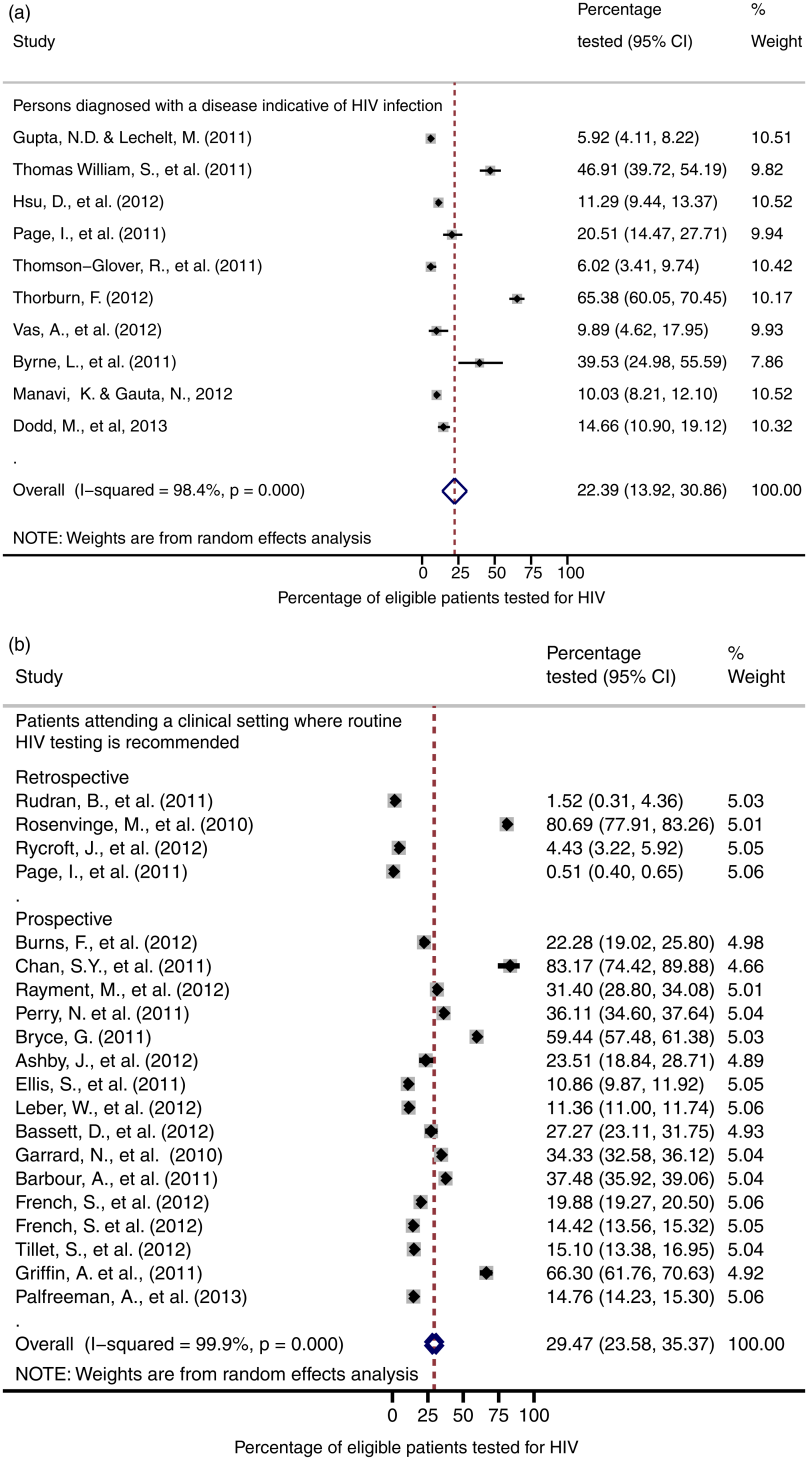
(A, B) Forest plots of percentage of eligible patients tested by group (A). Patients with an indicator disease (B). Patients attending a clinical setting where routine HIV testing is recommended (excluding genitourinary medicine/sexual health and antenatal clinics).

Table 1 shows the pooled estimates for the percentage of eligible patients who received HIV tests in the two groups, the percentage who were offered and accepted testing, and HIV prevalence from those studies that reported these outcomes. There was considerable heterogeneity within and between the patient groups, with an overall pooled estimate of 27.2% (95% CI 22.4% to 32.0%) of those eligible being tested. This level of heterogeneity is illustrated in the forest plots (figure 2A,B) for both groups. The higher pooled estimate of the two was 29.5% (95% CI 23.6% to 35.4%), with individual results ranging from 0.5% (95% CI 0.4% to 0.7%) in Page *et al*^11^ to 83.2% (95% CI 74.4% to 89.9%) in Chan *et al*^12^ (figure 2B). Testing was less likely in patients with diseases indicative of HIV infection, with a pooled estimate of 22.4% (95% CI 13.9% to 30.9%) and result ranging from 5.9% (95% CI 4.1% to 8.2%) in Gupta and Lechelt to 65.4% (95% CI 60.1% to 70.5%) in Thorburn *et al*^13^ There was considerable between-study heterogeneity across studies as illustrated by the I^2^ statistic value, which was consistently over 97%.

**Table 1:** Percentage of eligible patients who received HIV tests, plus percentages offered, accepted and HIV prevalence in those tested: summary results from random effects model meta-analysis patient group

Patient group	Percentage of those eligible who received HIV test (95% CI)	N studies	Percentage of those eligible who were offered HIV test (95% CI)	N studies	Percentage of those offered HIV test who accepted (95% CI)	N studies	Percentage of those tested who were HIV-positive (95% CI)	N studies
Patients diagnosed with indicator disease	22.4% (13.9% to 30.9%)	10	9.3% (1.2% to 17.3%)	2	87.4% (57.7% to 100%)	2	2.7% (1.1% to 4.4%)	6
Persons attending screening settings	29.5% (23.6% to 35.4%)	20	45.5% (28% to 63%)	12	69.2% (52.7% to 85.6%)	12	0.4% (0.2% to 0.6%)	17
Overall	27.2% (22.4% to 32%) I^2^=99.9%	30	40.4% (24.3% to 56.7%) I^2^=100%	14	71.5% (50% to 86.9%) I^2^=99.8%	14	0.5% (0.3% to 0.7%) I^2^=51.5%	23

95% CIs were bounded to between 0.00% and 100.00% as data are presented as a percentage. For test strategy and type of HIV test, some studies were excluded from the subgroup analyses due to lack of data.

A meta-regression was undertaken to explore other study characteristics that may contribute to the heterogeneity, and the results are presented in table 2. Covariates assessed as contributors to heterogeneity include location of testing (London or non-London), type of test administered (laboratory serological or point-of-care testing), testing strategy (opt-in or opt-out), service model (standard practice, staff training or GUM specialist testing) and study type (retrospective or prospective). None of these factors appeared to contribute significantly to the level of heterogeneity, and the results here should be interpreted with caution as many of the variables had small sample sizes, for example, there were only three studies in the ‘GUM specialist testing’ group of the service model. In a separate meta-regression model looking at contribution of study type in test coverage level exclusively in persons attending screening settings (the only group to have both retrospective and prospective study types), study type was found to be a significant contributor to the level of heterogeneity seen in test coverage in these studies (OR 6.3, 95% CI 1 to 38.4). The adjusted R^2^ for this meta-regression indicates that 15.1% of between-study variance in the pooled estimate for this testing across studies could be explained by study type in this group. The result here should again be carefully interpreted as only four studies were included in the ‘Retrospective’ group of this model.

**Table 2:** Predictors of HIV testing rates among eligible patients: meta-regression of results from studies identified

Covariate	N studies	OR (95% CI)	p Value
Patient group			
Patients presenting with indicator disease conditions	10	0.8 (0.2 to 2.6)	0.67
Persons attending screening settings	20	1 (ref)	
Location of study			
London	14	1 (ref)	
Non-London	16	0.5 (0.2 to 1.6)	0.26
Type of HIV test			
Laboratory	7	1 (ref)	
Point-of-care	6	0.8 (0.2 to 4)	0.75
Service model*			
Usual practice	18	1 (ref)	
Added staff training	9	1.3 (0.4 to 4.8)	0.62
GUM specialist testing	3	3.5 (0.5 to 24)	0.2
Testing strategy			
Opt-out	12	1 (ref)	
Opt-in	9	1 (0.3 to 3.2)	0.99
Study type			
Retrospective	14	1 (ref)	
Prospective	16	2.6 (0.9 to 7.7)	0.08

*With only three studies in one of the categories, this result should be interpreted with caution due to lack of power.

GUM, genitourinary medicine.

Fourteen studies reported both the number of tests being offered to those eligible and the number of those offered tests who were tested. The pooled estimate for HIV test offer level is at 40.4% (95% CI 24.3% to 56.7%) and the pooled estimate for HIV uptake levels is 71.5% (95% CI 56.0% to 86.9%). These results are presented in table 1 and show that the pooled estimate of eligible people offered an HIV test was 9.3% (95% CI 1.2% to 17.3%) in patients diagnosed with an indicator disease, which was lower than that seen in persons attending screening settings at 29.5% (95% CI 23.6% to 35.4%). Uptake, that is, the percentage of those offered testing who accepted was 69.2% (95% CI 52.8% to 85.6%) in persons attending screening settings and 87.4% (95% CI 57.7% to 100.0%) in patients diagnosed with indicator diseases, indicating that test offer is lower for patients with indicator diseases despite a higher test acceptance level in this group.

Of the 30 studies, 23 reported the number of those patients who tested positive for HIV, and the meta-analysis results for the seroprevalence observed in these studies are also presented in table 1. The pooled seroprevalence was 0.5% (95% CI 0.3% to 0.7%), with a higher seroprevalence seen in patients diagnosed with an indicator disease (2.7%, 95% CI 1.1% to 4.4%) than those tested in screening settings (0.4%, 95% CI 0.2% to 0.6%).

## Discussion

In this review, we found that the estimated percentage of patients eligible for HIV testing who receive a test is 27.2% (95% CI 22.4% to 32%). This low level of testing suggests that adherence to the 2008 UK guidelines for HIV testing is poor in recommended populations and settings. Analysis of test offer and acceptance levels suggests that the low overall level of testing is likely to be due to low levels of provider test offer and not patient acceptance. Provider test offer to those eligible was estimated to be only 40.4% (95% CI 24.3% to 56.7%) while patient acceptance of testing was 71.5% (95% CI 56% to 86.9%). This trend of low provider test offer and high-patient test acceptance has previously been seen in other countries in Europe and in the USA,^14^ ^15^ where it has been suggested that it indicates that health providers assess risk differently, are more likely to offer testing to patients they perceive to be at high risk or more likely to accept testing. Aside from this, operational and training barriers such as inadequate training for routine test offer, lack of time or difficultly in ordering an HIV test have also been cited as reasons contributing to low levels of health provider test offer.^16^^–^18

The highest level of testing (83.2%) was reported by Chan *et al,* who assessed the uptake and acceptability during a study of consecutive HIV test offer in medical admissions in Croydon. A previous audit of HIV testing in this hospital had showed a very low coverage of less than 1% prior to the prospective study. This indicates that consecutive test offer as undertaken in the prospective study can yield a much higher level of coverage. Cleary implementing a study of HIV offer is an intervention, and this may explain the significant difference in coverage in screening settings between retrospective audits and prospective studies. However, some retrospective studies also report high levels of testing, such as in Rosenvinge *et al*^19^ with 80.7%, indicating that good coverage can be achieved in the absence of a prospective study.

A higher HIV seroprevalence was found in patients tested who presented with a disease indicative of HIV infection at 2.7% (95% CI 1.1% to 4.4%) than found in those tested in settings where routine HIV testing should be undertaken 0.4% (95% CI 0.2% to 0.6%), and the overall pooled seroprevalence from studies was found to be 0.5% (95% CI 0.3% to 0.7%). These seroprevalence estimates exceed the threshold level 0.1% seropositivity of total tests administered deemed as cost-effective by CDC,^20^ indicating that HIV testing in these settings and populations is cost-effective and is likely to continue to be so with increased test coverage.

Of those presenting with indicator disease conditions (including tuberculosis, glandular fever and other blood borne viruses), an estimated 22.4% (95% CI 13.9% to 30.9%) received an HIV test compared with an estimated 29.5% (95% CI 23.6% to 35.4%) of those attending screening settings where routine HIV testing should be undertaken. Although the odds of being tested for HIV if diagnosed with an indicator disease condition do not appear to significantly contribute to the difference seen in test coverage (0.8, p=0.67), this group represents a particularly high-risk population who are easily identified. Testing in this group is a long-standing recommendation of guidelines prior to 2008, so these results are very disappointing. HIV testing in patients with indicator diseases has previously been explored. Read *et al*^21^ found that 37% of patients newly diagnosed with HIV in their secondary care hospital had presented to healthcare services with an HIV indicator condition in the preceding 12 months but had not been tested at the time. In a recent prospective study looking at the effectiveness of indicator condition-guided testing for HIV, Sullivan *et al*^22^ found an HIV prevalence of 1.8% (95% CI 1.42% to 2.34%) across European centres, similar to our estimate of 2.7% (95% CI 1.1% to 4.4%). Furthermore, findings from an analysis from 13 counselling and testing sites in Italy indicated that those presenting late with HIV were probably already infected at the time their initial indictor disease was diagnosed, but that there was a median lapse of 22.6 months between indicator disease diagnosis and HIV diagnosis.^23^ The lack of adherence to guidelines in this group therefore is likely to be hindering timely identification of HIV greatly.

There are a number of limitations to this study, primarily the lack of a comparable routine data set with relevant information. Due to this we have been reliant on a relatively small number of reports from local audits and studies that included a wide variety of populations, settings, duration and methods used for measuring HIV testing. However, as guideline recommendations are broad in their description of settings and populations, further restriction in inclusion criteria was not possible. The studies were of varied quality, and this could not be systematically assessed as many were published as reports or conference abstracts rather than peer-reviewed papers. Data quality was also variable, with some dependent on patient self-report of previous tests to define eligibility. Several studies were interventional in nature, offering consecutive HIV tests in recommended settings and this may have contributed to an overestimate of testing in routine conditions. However, these limitations could only be overcome through the implementation of standards for reporting in the context of some surveillance system such as those that already exist in established testing settings.

There was a great deal of heterogeneity in the data with some I^2^ statistic values at 100%, and as a result caution should be taken in interpreting the summary statistics presented for illustration as an average proportion. True study percentages are likely to vary greatly around the estimate points presented. We do not claim to present a true level of overall test coverage level but rather an estimate from the data collected and we have tried to understand some of the variation that was associated with this. Meta-regression did not identify any factor as the majority a contributor to the between-study variance seen, and it is likely that much more of the heterogeneity could be explained by factors that could not be measured in the meta-regression either due to insufficient study numbers or the fact that potential explanatory variables were not reported for all studies.

## Conclusion

The results of this review and meta-analysis indicate adherence to 2008 national guidelines for HIV testing in the UK is poor and that low levels of provider test offer appear to be a major contributor to this, particularly in patients presenting with an indicator disease. Failure to adhere to testing guidelines is likely to be contributing to late diagnosis with implications for poorer clinical outcomes and continued onwards transmission of HIV. Improved surveillance of HIV testing outside of specialist settings may be useful in increasing adherence testing guidelines.

**Key messages**

- Findings indicate HIV test coverage in the UK is poor and low levels of provider test offer appear to be the main contributor to this.
- Seroprevalence estimates show that HIV testing is cost-effective and increasing HIV testing in line with national guidelines would also be cost-effective.
- Further exploration of effective methods for the routine offer of HIV testing in recommended settings.
- Better methods for the clear dissemination of routine HIV testing messages to non-specialist clinicians are required.

## Supplementary Material

Web supplement
