# Supplementary material for: Low levels of HIV test coverage in clinical settings in the UK: a systematic review of adherence to 2008 guidelines
Source: Sex Transm Infect. 2014 Jan 10;90(2):119–24. doi: 10.1136/sextrans-2013-051312 (PMC3945742; doi:10.1136/sextrans-2013-051312)
Supplement: Web supplement [file sextrans-2013-051312-s1.pdf]

Appendix a: Classification of settings and populations for routine HIV testing (adapted from national testing guidelines, BHIVA 2008) [6]

**Persons diagnosed with a disease indicative of HIV infection**	Tuberculosis<br>Pneumocystis<br>Cerebral toxoplasmosis<br>Primary Cerebral Lymphoma<br>Cryptococcal meningitis<br>Progressive multifocal leucoencephalopathy<br>Kaposi's sarcoma<br>Persistent cryptosporidiosis<br>Non-Hodgkin's Lymphoma<br>Cervical Cancer<br>Cytomeglovirus retinitis<br>Bacterial pneumonia<br>Aspergillosis<br>Aseptic meningitis/encephalitis<br>Cerebral abscess<br>Space occupying lesion of unknown cause<br>Guillain-Barré syndrome<br>Transverse myelitis<br>Peripheral neuropathy<br>Dementia<br>Leucoencephalopathy<br>Severe or recalcitrant seborrhoeic dematitis<br>Severe or recalcitrant psoriasis<br>Multidermatomal or recurrent herpes zoster<br>Oral candidiasis<br>Oral hairy leukoplakia<br>Chronic diarrhoea of unknown cause<br>Weight loss of unknown cause<br>Salmonella, shigella or campylobacter<br>Hepatitis B infection<br>Hepatitis C infection<br>Anal cancer or anal intraepithelial dysplasia<br>Lung cancer<br>Seminoma<br>Head and neck cancer<br>Hodgkin's lymphoma<br>Castleman's disease<br>Vaginal intraepithelial neoplasia<br>Cervical intraepithelial neoplasia (Grade 2 or above)<br>Any unexplained blood dyscrasia including:<br>Thrombocytopenia<br>Neutropenia<br>Lymphopenia<br>Infective retinal disease including herpesviruses and toxoplasma<br>Any unexplained retinopathy<br>Lymphadenopathy of unknown cause<br>Chronic parotitis<br>Lymphoepithelial parotid cysts<br>Mononucleosis-like syndrome (primary HIV infection)<br>Pyrexia of unknown origin<br>Any lymphadenopathy of unknown cause<br>Any sexually transmitted infection
**Persons attending a service where routine HIV screening is undertaken**	Termination of pregnancy services<br>Drug dependency programmes<br>All patients presenting for healthcare where HIV prevalence

**(excluding GUM and antenatal services)**	All men and women registering in general practice where diagnosed HIV prevalence in the local population exceeds 2 in 1000 population<br>All general medical admissions where diagnosed HIV prevalence in the local population exceeds 2 in 1000 population

Appendix b: Characteristics of Studies Included: Methods, Measures and Testing Levels

Author	Risk group	Setting (diagnosed HIV prevalence per 1,000 population 15-59 year olds) *	Methods	Number eligible to test	Number offered test	Number tested	Number testing positive
Persons diagnosed with a disease indicative of HIV infection							
Gupta, N.D. & Lechelt, M. (2011) [1]	Inpatients with indicator diseases (tuberculosis, hepatitis B, hepatitis C, cervical intraepithelial neoplasia (grade I/II), lymphoma, anal cancer, seminoma, aspergillosis or Castleman's disease)	South-west Essex (1.28)	Electronic record audit of attendees attending one secondary care hospital	557	33	33	Not reported
Thomas William, S., *et al.* (2011) [2]	Patients with indicator disease (tuberculosis)	Birmingham and Solihull (Birmingham East & North; 1.5, Heart of Birmingham; 3.29, South Birmingham; 1.66; Solihull; 0.58)	Retrospective audit	194	Not reported	91	Not reported
Hsu, D., *et al.* (2012) [3]	Primary care patients presenting with glandular fever-like illness	South London (Lambeth 13.28, Southwark; 10.29)	Retrospective audit of patients attending 72 primary care clinics	1045	Not reported	118	3
Page, I., *et al.* (2011) [4]	Patients with indicator disease (tuberculosis, hepatitis B, hepatitis C, lymphoma)	Blackpool (3.41)	Retrospective audit of patients attending one secondary care hospital	156	Not reported	32	Not reported
Thomson-Glover, R., *et al.* (2011) [5]	Patients with indicator disease (hepatitis B, hepatitis C, candida	Warrington (0.6)	Case-note audit of patients attending two secondary care hospitals	249	Not reported	15	0

	stomatitis)						
Thorburn, F. (2012) [6]	Patients with indicator disease (diagnosed with tuberculosis)	Glasgow (1.7)	Retrospective case-note review of TB patients attending one tertiary care clinic	338	Not reported	221	9
Vas, A., *et al.* (2012) [7]	Patients with indicator disease (tuberculosis, hepatitis B, hepatitis C)	Manchester (5.22)	Retrospective case-note review of patients attending one secondary care hospital	91	13	9*	Not reported
Byrne, L., *et al.* (2011) [8]	Patients admitted to acute medical unit with community-acquired pneumonia	London (Newham; 8.12, Tower Hamlets; 5.94)	Retrospective case-note review of patients attending one acute medical admissions unit	43	Not reported	17	2
Manavi, K., Gautam, N. (2012) [9]	Patients diagnosed with clinical indicator conditions as specified in UK HIV testing guidelines	Birmingham (3.29)	Retrospective case note review of patients attending one secondary care hospital	967	Not reported	97	1
Dodd, M. et al (2013) [10]	Patients with an HIV indicator illness in the presenting complaint or past medical history.	Sheffield (1.4)	Retrospective case note review of patients in one General Intensive Care Unit	307	Not reported	45	3
Persons attending recommended testing settings in high prevalence areas							
Burns, F., *et al.* (2012) [11]	Acute medical admissions	London (5.24)	Prospective, consecutive HIV test offer to patients attending one acute medical admissions unit	606	282	135	3
Chan, S.Y., *et al.* (2011) [12]	Acute medical admissions	Croydon (4.45)	Prospective offer of HIV test to patients attending one acute medical admissions unit	101	101	84	0
Rayment, M., *et al.* (2012) [13]	Acute Care unit and Dermatology outpatient clinic	London (City and Hackney (8.25)	Prospective study of patients attending one acute care unit	1223	548	384	4
Perry, N., *et al.* (2011) [14]	Acute medical admissions	Brighton & Hove PCT (7.57)	Prospective HIV test offer to patients attending one acute medical admissions unit	3913	1553	1413	2
Bryce, G., (2009) [15]	Patients newly registering with GP	Brighton & Hove PCT (7.57)	Prospective HIV test offer to patients attending nine primary	2478	Not reported	1473	2

			care clinics				
Ashby, J., *et al.* (2012) [16]	Polyclinic attendees in high prevalence area	West London (Kensington & Chelsea; 8.3, Hammersmith & Fulham; 8.5, Westminster; 7.01)	Prospective study of patients attending one polyclinic	302	93	71	0
Ellis, S., *et al.* (2011) [17]	Acute medical admissions	Newcastle Upon Tyne (1.61)	Prospective audit of patients attending one acute medical admissions unit	3645	478	396	2
Rudran, B., *et al.* (2011) [18]	Acute medical admissions	Bournemouth and Poole (2.32)	Retrospective case-note review of patients attending one acute medical admissions unit	198	3	3	Not reported
Leber, W., *et al.* (2012) [19]	Patients newly registering with GP	Hackney (8.25)	Cluster randomised control trial of patients attending 40 primary care units	28274	6607	3213	7
Bassett, D., *et al.* (2012) [20]	Acute medical admissions	Manchester (5.22)	Prospective case-note review of patients attending one acute medical admissions	429	134	117	Not reported
Rosenvinge, M., *et al.* (2010) [21]	Women attending termination of pregnancy services	Wandsworth (4.91)	Retrospective review of HIV testing of patients who attended two termination of pregnancy clinic	870	844	702	1
Garrard, N., *et al.* (2010) [22]	Women attending termination of pregnancy service	Southwark (10.39) and Lambeth (13.28)	Prospective, consecutive test offer to patients attending one termination of pregnancy clinic	2,831	Not reported	972	5
Barbour, A., *et al.* (2012) [23]	Patients admitted to acute medical admissions	Croydon (4.45)	Prospective intervention at one acute medical unit	3709	Not reported**	1390	7
Rycroft, J., *et al.* (2012) [24]	Acute medical admissions	Greenwich (5.58)	Retrospective audit of patients who attended one acute medical admissions	970	Not reported	43	3
Page, I., *et al.* (2011) [4]	Acute medical admissions	Blackpool (3.41)	Retrospective audit of patients who attended one secondary care hospital	13,999	Not reported	72	Not reported

French, S., *et al.* (2012) [25]	Patients newly registering with GP	Southwark (10.39), Lewisham (7.03), Lambeth (13.28)	Prospective study of patients attending 13 primary care clinics	16,241	6405	3229	12
French, S., *et al.* (2012) [25]	Patients newly registering with GP	Southwark (10.39), Lewisham (7.03), Lambeth (13.28)	Prospective study of patients attending 5 primary care clinics	6275	4925	905	11
Tillet, S., *et al.* (2012) [26]	Acute medical admissions	Tower Hamlets (5.94)	Prospective study of patients attending one secondary care hospital	1596	Not reported	241	2
Griffin, A., *et al.* (2011) [27]	Patient newly registering with GP	Manchester (5.22)	Prospective study of patients attending one primary care clinic	457	Not reported	303	2
Palfreeman, A., *et al.* (2013) [28]	Patients attending admitted to AMU	Leicester (3.22)	Prospective study of patients admitted to AMU	17226	Not reported	2542	29

* reported for patients diagnosed with TB only. ** 77 test refusals were reported. Additional data regarding testing strategy (opt-in vs. opt-out), service model (standard care vs. specific staff training vs. GUM/Health advisor-led testing), and type of HIV test (POCT vs. 4th generation serology).

Appendix c: Supplementary data tables for studies identified by group

Study	Title	Article	Primary testing outcome	Exclusions	Time period (duration)	Population	Number of centres	Type of centre	Measure/reporting method
**Persons diagnosed with a disease indicative of possible HIV infection**									
**Gupta, N.D. & Lechelt, M. (2011)**	**Assessment of the implementation and knowledge of the UK national guidelines for HIV testing (2008) in key conditions at**	Yes - Audit Report	Electronic departmental record or HIV testing and Electronic pathology records	Non-verifiable data	August 2009 – June 2012 (11 months)	Inpatients at Basildon & Thurrock Hospital	1	Secondary care hospital	Electronic record of HIV test

	**a UK district general hospital**								
**Thomas William, S., *et al.* (2011)**	**Changes in HIV testing rates among patients with tuberculosis in a large multi-ethnic city in the UK**	Yes - Audit Report	Laboratory database record of HIV test	<18 years, private patients, chemoprophylaxis patients, non-tuberculosis mycobacteria, diagnosis outside catchment area	September 2008 – March 2009 (6 months)	Patients registered on the Birmingham Tuberculosis aftercare register	>1	Various	Laboratory record of HIV test
**Hsu, D., *et al.* (2012)**	**Diagnosing HIV infection in patients presenting with glandular fever-like illness in primary care: are we missing primary HIV infection?**	Yes - Short Communication	Record of HIV test	Not reported	April 2009 - June 2010 (14 months)	Primary care patients in Lambeth and Southwark	72	Primary care clinics	Laboratory record of HIV test request
**Page, I., *et al.* (2011)**	**The impact of new national HIV testing guidelines at a district general hospital in an area of high HIV seroprevalence**	Yes - Paper	Laboratory record of HIV test	HIV test requests from GUM clinics	October 2008 – September 2009	Inpatients in Blackpool	1	Secondary care hospital	Laboratory record of HIV test

**Thomson-Glover, R., *et al.* (2011)**	**Diagnosing HIV in non-GUM secondary care settings**	No - Abstract	Record of HIV test	not reported	November 2009 - April 2010 (6 months)	inpatients Warrington & Halton hospitals	2	Secondary care hospitals	Record of HIV test
**Thorburn, F. (2012)**	**The impact of a multi-disciplinary meeting on the rates of HIV in testing in TB patients**	No - Abstract	Record of HIV test offer	not reported	2010 - 2011 (duration not reported)	Tuberculosis patients attending virology centre in Glasgow	1	Tertiary care clinic	Laboratory record of HIV test administration
**Vas, A., *et al.* (2012)**	**HIV testing and in TB and Hepatitis services in a district general hospital**	No - Abstract	Record of HIV test	not reported	2009 (duration not reported)	Indicator disease patients in a Manchester hospital	1	Secondary care hospital	Record of HIV test administration
**Byrne, L., *et al.* (2011)**	**HIV specialists must lead the way to make HIV testing truly routine**	No-Abstract	Record of HIV test	, <18, non-medical specialty, underlying chronic lung disease, hospital-acquired pneumonia	February - April 2010 (3 months)	Patients admitted with community-acquired pneumonia	1	Acute medical admissions unit	Case-note record of HIV test administration
**Persons attending services where routine HIV screening should be undertaken**									
**Chan, S.Y., *et al.* (2011)**	**Acceptance of HIV testing in medical inpatients: A local acceptability study**	Miscellaneous	Consent to have an HIV test	<15 and >59 years, total time admitted <24 hours, assessed as unable to consent, known to be HIV-positive	September 2009 (2 weeks)	Acute medical admissions in Croydon	1	Acute medical admissions unit	Offer and acceptance of HIV test

**Rayment, M., *et al.* (2012)**	**HIV Testing in Non-Traditional Settings - the hints study: A multi-centre observational study of feasibility and acceptability.**	Article	Offer of HIV test to eligible individual	<16, >65 years, known HIV positive, not accessing healthcare for the first time in testing period, not able to consent	January – September 2010 (12 weeks each site)	Patients attending primary and secondary healthcare services in 4 London centres	4	Acute care units, Dermatology OPD	Administration of HIV oral fluid or 4th generation HIV serology
**Perry, N. *et al.* (2011)**	**HIV testing in acute general medical admissions must be universally offered to reduce undiagnosed HIV**	Abstract	Record of HIV test	<16 and >79 years, known HIV positive	August 2009 – January 2010 (5 months)	Acute medical admissions in Brighton	1	Acute medical admissions unit	HIV test result
**Bryce, G. (2009)**	**A study to assess the acceptability, feasibility and cost-effectiveness of universal HIV testing with newly registering patients (aged 16-59) in primary care**	Abstract	Acceptance of HIV test offer	<16 and >59 years	May - November 2010 (4 months)	Patients attending primary care services in Brighton	9	Primary care clinics	HIV POCT test
**Ashby, J., *et al.* (2012)**	**HIV testing uptake and acceptability in an inner city polyclinic**	Article	Acceptance of HIV test offer	<16, >65 years, unable to consent	2011 dates not specified (random 4-hour duration over a 4 week period)	Polyclinic attendees in west London	1	Polyclinic	Rapid point-of-care HIV test

**Ellis, S., *et al.* (2011)**	**Offering HIV testing in an acute medical admissions unit in Newcastle upon Tyne**	Clinical Medicine research	Record of HIV test	<18 years, no capacity for consent	September - October 2009 (11 weeks) and January - March 2010 (6 weeks)	Acute medical admissions in Newcastle	1	Acute medical admissions unit	HIV test offer and administration
**Rudran, B., *et al.* (2011)**	**HIV testing in acute medical admissions**	Abstract	Record of HIV test	not reported	2010 - exact date not specified (1 week)	Acute medical admissions in Bournemouth	1	Acute medical admissions unit	Hospital or laboratory database record of HIV test
**Leber, W., *et al.* (2012)**	**Can point-of-care HIV testing in primary care increase identification of HIV? The RHIVA 2 Cluster randomised control trial - update**	Abstract	Offer of rapid point-of-care HIV test	<16 years,	May 2010 end date not specified	Patients attending primary care services in London	40	Primary care units	Administration of rapid point-of-care HIV test
**Bassett, D., *et al.* (2012)**	**Practical challenges implementing national HIV testing guidelines in general medical admissions**	Abstract	Record of HIV test	not reported	July 2011 (two weeks)	Acute medical admissions in central Manchester	1	Acute medical admissions	Record of HIV test administration

**Rosen vinge, M., *et al.* (2010)**	**A successful uptake of HIV testing in south London termination of pregnancy services**	Abstract	Record of consent for an HIV test	Known HIV positive, recent (< 6 months) HIV negative test, repeat attendance	April - December 2009 (9 months)	Women attending termination of pregnancy services in south London	2	Termination of pregnancy clinics	Paper and electronic record of HIV test administration
**Garrar d, N., *et al.* (2010)**	**Opt-out HIV testing pilot in termination of pregnancy services - 11-month service evaluation**	Abstract	HIV test recommend ation	not reported	November 2008 - September 2009 (11 months)	Women attending termination of Pregnancy services north London	1	Termination of pregnancy clinic	Documentation of HIV test result
**Barbo ur, A., *et al.* (2011)**	**Opt-out HIV testing policy implemented as routine standard of care for acute medical admissions in a high prevalence area**	Abstract	Record of HIV test	<16 and >79 years,	July 2011 – December 2011 (6 months)	Patients attending acute medical admissions in Croydon	1	Acute medical admissions	HIV testing
**Rycrof t, J., *et al.* (2012)**	**HIV testing in the acute medical unit - setting the scene for universal opt-out testing**	Abstract	Laboratory record of HIV test	Not reported	June & November 2011 (audited 2 weeks for each admissions cycle)	Patients attending acute medical admissions in south east London	1	Acute medical admissions	Record of HIV test in laboratory database
**Page, I., *et al.* (2011)**	**The impact of new national HIV testing guidelines at a district general hospital in an area of high**	Paper	Laboratory record of HIV test	HIV test requests from GUM clinics	October 2008 – September 2009	Inpatients in Blackpool	1	Secondary care hospital	Laboratory record of HIV test

	**HIV seroprevalence**								
**Palfreeman, A., et al. (2013)**	**HIV testing for acute medical admissions: evaluation of a pilot study in Leicester, England**	Paper	Laboratory record of HIV test	Not reported	September 2008 – August 2011 (36 months)	Patients admitted to AMU in Leicester	1	Secondary care hospital	Laboratory record of HIV test

References

1. Gupta ND, Lechelt M. **Assessment of the implementation and knowledge of the UK national guidelines for HIV testing (2008) in key conditions at a UK district general hospital.** International Journal of STD and AIDS 2011;22(2):102-04
2. Thomas William S, Taylor R, Barrett S, et al. **Changes in HIV testing rates among patients with tuberculosis in a large multi-ethnic city in the UK.** International Journal of STD and AIDS 2011;22(12):748-50
3. Hsu DTS, Ruf M, O'Shea S, et al**. Diagnosing HIV infection in patients presenting with glandular fever-like illness in primary care: are we missing primary HIV infection?** HIV Medicine 2013;14(1):60-63
4. Page I, Phillips M, Flegg P, et al. **The impact of new national HIV testing guidelines at a district general hospital in an area of high HIV seroprevalence.** Journal of the Royal College of Physicians of Edinburgh 2011;41(1):9-12
5. Thomson-Glover DM, Smalley L. **Diagnosing HIV in non-GUM secondary care settings.** HIV Medicine 2011;12(1):14-86
6. Thorburn F. **The impact of a multi-disciplinary meeting on the rates of HIV in testing in TB patients.** HIV Medicine 2012;13(1):1-11
7. Vas A, Morgan E, Padmankumar K, et al. **HIV testing in TB and Hepatitis services in a district general hospital.** HIV Medicine 2012;13(1):1-11
8. Byrne L, Whitburn T, Vearncombe S, et al. **HIV specialists must lead the way to make HIV testing truly routine.** HIV Medicine 2011;12(1):14-86
9. Manavi K, Gautam N. **Does identification of patients with HIV clinical indicator diseases lead to offer of HIV testing?** Evidence and resources to commission expanded HIV testing in priority medical services in high prevalence areas, Health Protection Agency 2012.
10. Dodd MC, Collini PJ, Dockrell DH. **Low concordance with HIV testing guidelines in a retrospective review of intensive care practice.** Thorax 2013;68(11);1072-4
11. Burns F, Edwards SG, Woods J, et al. **Acceptability and Feasibility of Universal Offer of Rapid Point of Care Testing for HIV in an Acute Admissions Unit: Results of the RAPID Project.** PLoS One 2012;7(4)
12. Chan SY, Hill-Tout R, Rodgers M, et al. **Acceptance of HIV testing in medical inpatients: a local acceptability study.** International Journal of STD & AIDS 2011;22(4):187-89
13. Rayment M, Thornton A, Mandalia S, et al. **HIV Testing in Non-Traditional Settings – The HINTS Study: A Multi-Centre Observational Study of Feasibility and Acceptability.** PLoS One 2012;7(6):e39530
14. Perry N, Heald L, CassellL J, et al. **HIV testing in acute general medical admissions must be universally offered to reduce undiagnosed HIV.** Health Protection Agency. Time to Test for HIV: Expanding HIV testing in healthcare and community services in England, 2011
15. Bryce N, Jeffery M, Hankins M, et al. **A study to assess the acceptability, feasibility and cost-effectiveness of universal HIV testing with newly registering patients (aged 16-59) in primary care.** Health Protection Agency. Time to Test for HIV: Expanding HIV testing in healthcare and community services in England, 2011
16. Ashby J, Braithewaite B, Walsh J, et al. **HIV testing uptake and acceptability in an inner city polyclinic.** AIDS Care 2012;24(7):905-09
17. Ellis S, Graham L, Price DA, Ong ELC. **Offering HIV testing in an acute medical admissions unit in Newcastle upon Tyne.** Clinical Medicine, Journal of the Royal College of Physicians of London 2011;11(6):541-43
18. Rudran B, Jarvis M, Thomas D, et al**. HIV testing in acute medical admissions.** HIV Medicine 2011;12(1):14-86
19. Leber W, McMullen H, Bremner S, et al. **Can point of care HIV testing in primary care increase identification of HIV? The RHIVA 2 cluster randomised controlled trial - update.** HIV Medicine 2012;13(1):1-11

20. Bassett D, Cousins D, Davies TL, et al. **Practical challenges implementing national HIV testing guidelines in general medical admissions.** HIV Medicine 2012;13(1):1-11
21. Rosenvinge M, Majewska W, Valcarcel E, et al. **A successful uptake of HIV testing in south London termination of pregnancy services.** HIV Medicine 2010;11(s1):1-119
22. Garrard N, Peck J, Ruf M, et al**. Opt-out HIV testing pilot in termination of pregnancy services - 11-month service evaluation.** HIV Medicine 2010;11(s1):1-119
23. Barbour A, Philips S, Draper S, et al. **Opt-out HIV testing policy implemented as routine standard of care for acute medical admissions in a high prevalence area: effective and sustainable.** HIV Medicine 2012;13(1):1-11
24. Rycroft J, Hall R, Kegg S. **HIV testing in the Acute Medical Unit - setting the scene for universal opt-out testing.** HIV Medicine 2012;13(1):13-85
25. French S, Vieu MN, Peck J, et al. **Expanding new patient HIV testing in primary care in Lambeth, Southwark and Lewisham (LSL). Expanding access: HIV testing in extended settings.** Health Protection Agency. Time to Test for HIV: Expanding HIV testing in healthcare and community services in England, 2011
26. Tillet S, Orkin C, Nori A. I**ntroducing opt-out HIV testing in the Acute Admissions Unit: Experience of the first 2 months.** Expanding access: HIV testing in extended settings. PHAST, 2012.
27. Griffin A, Sarwar S, Shelton R, et al. **HIV, Hepatitis B and C testing in primary care.** Expanding access: HIV tesitng in extended settings. PHAST, 2012
28. Palfreeman A, Nyatsanza F, Farn H, et al. **HIV testing for acute medical admissions: evaluation of a pilot study in Leicester, England.** Sexually Transmitted Infections 2013;89(4):308-10
